# Rapid and recent evolution of fetA phase variability among hyperinvasive MenW:cc11 disease isolates

**DOI:** 10.1099/mgen.0.001655

**Published:** 2026-02-20

**Authors:** Mercy W. Kimani, Jack L. Clark, Luke R. Green, Christopher D. Bayliss

**Affiliations:** 1 Division of Microbiology and Infection, School of Biological and Biomedical Sciences, College of Life Sciences, University of Leicester, Leicester, UK; 2 School of Clinical Dentistry, University of Sheffield, Sheffield, UK

**Keywords:** *Neisseria*, meningococcus, phase variation, *fetA*, MenW, pandemic

## Abstract

*Neisseria meningitidis* serogroup W (MenW) clonal complex 11 isolates are a major worldwide cause of invasive meningococcal disease. This lineage has exhibited significant plasticity with multiple evolutionary events occurring during international and national spread. Phase variation is a major mechanism for the modulation of surface antigen expression including for FetA, a major immunogenic meningococcal antigen. Alterations in switching rates occur due to changes in the repeat numbers of phase-variable genes. Analysis of 1521 MenW:cc11 carriage and disease isolates detected evidence of ongoing evolution within the UK. The rebound in MenW:cc11 disease in the post-coronavirus disease 2019(COVID-19) pandemic era was associated with isolates belonging to four sub-clusters (SC). One of these SCs was associated with isolates circulating at low levels for more than 50 years, whilst another contained novel variants. Evolution of the *fetA* promoter and repeat region was detected with increases in the tract length from 6C to 8C or longer becoming highly prevalent from 2015 onwards. Acquisition of phase-variable tracts of 8C or longer was correlated with other alterations in the *fetA* promoter and genic region. The predominant repeat number for this promoter-located repeat tract was 11C, which is associated with intermediate expression. The potential for heightened phase variation of FetA mirrors earlier evolution of increases in repeat numbers of two other surface proteins, PorA and Opa, in this lineage. Ongoing evolution of phase variation may be aiding maintenance of significant levels of the transmission of this hypervirulent lineage and persistent associations with invasive disease despite widespread vaccine usage.

**Impact Statement**

*Neisseria meningitidis* is a major cause of meningitis and septicaemia worldwide. Due to frequent disease association, several *N. meningitidis* lineages are classified as hyperinvasive with one, serogroup W clonal complex 11, spreading worldwide over the last 25 years. This lineage exhibits high levels of genetic plasticity. We have observed that post-COVID-19 pandemic invasive isolates of this lineage occurred in a small number of genetically related subclusters containing pre-existing and novel variants. Multiple genes of this pathogen are subject to high-frequency alterations in gene expression, termed phase variation, due to hypermutable repetitive DNA tracts. Starting in 2015, isolates of this lineage evolved the ability to undergo phase variation of FetA, a major surface antigen, with 25% of post-pandemic isolates having this capacity. This heightened capacity to stochastically alter the expression of this major surface antigen may facilitate ongoing immune evasion, transmission and disease by this hyperinvasive *N. meningitidis* strain.

## Data Summary

The nanopore sequences and hybrid genome sequences are available in the EMBL-EBI ENA database with the accession numbers listed in Data File S1 (available in the online Supplementary Material). The hybrid genome sequences are also available on the Neisseria PubMLST database with the identification numbers listed in Data File S1. The aligned sequences for the pangenomes, *csw*, *fetA* (nucleotide and amino acid) and *fetA* promoter are available in the Microbiology Figshare repository (https://doi.org/10.6084/m9.figshare.30706913)[1].

## Data Availability

This publication made use of the Neisseria PubMLST website (https://pubmlst.org/neisseria/), by the University of Oxford and developed with funding from the Wellcome Trust and European Union (19), which encompasses the Meningitis Research Foundation Meningococcus Genome Library (https://www.meningitis.org/global-meningitis-genome-library/) initially developed by Public Health England (now UKHSA), the Wellcome Trust Sanger Institute and the University of Oxford as a collaboration, and whose initial project was funded for 3 years by the Meningitis Research Foundation. This publicly available database contains the genome sequences and gene sequences of all isolates reported herein.

## Introduction

Meningitis and septicaemia arising from invasive infections by *Neisseria meningitidis* (also called the meningococcus), a Gram-negative diplococcus, are major causes of morbidity and mortality particularly among infants, teenagers and young adults. Hyperinvasive lineages of *N. meningitidis*, such as those of the genogroup W clonal complex 11 (MenW:cc11) lineage, are major causes of meningitis and septicaemia worldwide. The capsular type or genogroup (indicating presence of the capsular biosynthetic genes) is a major virulence determinant with the W capsule being one of six capsule structures associated with most cases of invasive disease by this bacterial pathogen. Asymptomatic carriage in teenagers and young adults is a critical driver of the spread and evolution of all meningococcal lineages with evolvability facilitated by a plethora of mechanisms for rapid generation and lateral gene transfer of genetic and antigenic variation.

Phase variation (PV) is a significant source of both phenotypic and antigenic variation for the meningococcus generating high-frequency reversible switches in the expression of outer membrane proteins (OMPs) and other surface determinants [2]. Meningococcal PV is mediated by simple sequence repeat (SSR) tracts, which undergo high rates of deletions or insertions during DNA replication. SSR within the ORF results in on/off switches in gene expression (i.e. translational PV), whilst those within promoter regions mediate variations in expression (i.e. transcriptional PV). Approximately 40 genes per isolate are subject to SSR-mediated PV in meningococci due mainly to polyG or polyC tracts but also tetra-, penta- and hepta-nucleotide repeats [3]. Evolution of heightened PV was previously observed among UK MenW:cc11 isolates for PorA and Opa, major surface OMPs [4]. A recent phenotype-to-genotype analysis of 167 UK MenW:cc11 isolates, obtained between 2009 and 2016, indicated that a capsular gene, *csw*, and PV of the ferric enterochelin protein (FetA) were key determinants of disease- or carriage-associated phenotypes [5]. PV of *fetA* was also a determinant of disease/carriage status as PV of this major antigenically variable OMP was only detected in carriage isolates. This was unusual as *fetA* is subject to PV in invasive meningococcal disease (IMD) isolates of most meningococcal lineages.

Meningococcal strains are classified based on phylogenetic groupings with lineages referring to genetically related isolates with the same sequence type or groups of closely related sequence types termed clonal complexes. The cc11 lineage was first associated with endemic and epidemic cases of IMD in the 1960s and had a genogroup C capsule. MenW:cc11 emerged in the 1970s following two recombination events in the *cps* locus that led to a capsular switching event from MenC to MenW [6]. Following MenW:cc11 epidemics in Mecca during the 2000 Hajj pilgrimage, this lineage has spread globally to cause epidemics in the meningitis belt of Sub-Saharan Africa and become endemic in Europe, Asia and South America [7]. Over this period, the MenW:cc11 lineage underwent significant divergence to form the Hajj and South American sublineages, with the latter spreading to and becoming endemic in Brazil, Argentina and the UK [8]. This sublineage evolved further to generate the novel 2013 strain, which rapidly became the leading cause of MenW IMD cases in the UK, and is characterized by seven genomic changes, including one in *csw* [9]. These genetic evolutionary steps may have facilitated the ongoing spread and disease contributions of this hypervirulent meningococcal lineage.

During the coronavirus disease 2019 (COVID-19) pandemic, major reductions in IMD occurred in many countries around the world with 17 out of 21 countries undergoing a drop in IMD cases during the first lockdown period (March–May 2020) as compared to the same periods in 2018 and 2019 [10]. The UK, for example, experienced a 73% reduction in IMD case rates following the March 2020 lockdown [11]. A post-pandemic rebound has resulted in IMD cases returning to or even exceeding pre-pandemic levels in multiple countries [12]. This rebound has been associated with significant shifts in the prevalence of certain strains, for example, a sharp rise in UK IMD from September 2021 was associated with an increase in MenB cases from 57% pre-pandemic to 89% post-pandemic [13]. Meningococcal transmission routes may also have changed as the proportion of IMD cases in 18–24 year olds in the UK increased from 13 to 38% before and after the pandemic, respectively [13].

Polysaccharide capsules are required for meningococcal virulence and are widely utilized for typing with serogroup indicating expression of a specific capsule whilst genogroup indicates the presence of the biosynthetic genes within the chromosome. Genes involved in capsule synthesis are clustered at a single locus in the meningococcal genome, named *cps*. The *cps* locus comprises six regions, labelled A, B, C, D, D’ and E, with the order of these regions conserved across serogroups. Genes within these regions are also conserved with the exception of region A genes, which encode the enzymes responsible for capsule synthesis [14]. In MenW, region A comprises seven genes, four of which are conserved across serogroups B, C, W and Y, and another two genes are conserved between MenW and MenY. The remaining gene, *csw*, is the principal determinant of MenW capsule structure and encodes a chimeric, monomeric protein with two active sites that have sialyltransferase and galactosyltransferase activities [15]. This protein is responsible for linking alternating molecules of d-galactose and sialic acid to produce the MenW capsule.

The *fetA* gene is subject to transcriptional PV due to a polyC tract located in the core promoter region [16]. FetA, previously FrpB, is an iron-regulated TonB-dependent receptor for enterobactin, a siderophore, and is present in the outer membranes of nearly all carriage and invasive meningococcal isolates [17]. An immunodominant, externally exposed loop in this protein is encoded by a variable region (VR) that has been used for meningococcal subtyping and may be a critical determinant of strain-specific protective OMV vaccine responses [18,19].

Although MenW-associated cases have declined in the UK, MenW:cc11 remains an important cause of IMD. Using the entire UK collection of MenW:cc11 whole-genome sequences on the *Neisseria* PubMLST database, we investigated the overall post-pandemic population structure and allelic changes in two key genes: *csw*, the major virulence determinant, and *fetA*, a major immunogen. We also assessed whether PV of *fetA* was either not required for IMD by MenW:cc11 isolates or that the MenW:cc11 lineage was undergoing another novel evolutionary event. Our results show that four novel subclusters (SCs) drove most pandemic and post-pandemic MenW:cc11 IMD in the UK and that some of these lineages are associated with a shift in the length of the *fetA* SSR, indicative of heightened propensity for PV of this major meningococcal antigen, adding to the previously reported heightened propensity for PV of other OMPs in this lineage.

## Methods

### Genome data

The *Neisseria* PubMLST database had 1,827 UK MenW:cc11 isolates with 1,759 whole-genome sequences submitted as of November 2024. Replicate isolates (either used for validating sequencing platforms or in different studies) and closed genomes (to be used as reference genomes) were removed, leaving 1,521 isolates for analyses (Data File S1). The 11 reference genome sequences consisted of a single contig and were generated by combining nanopore and Illumina sequence data (Green *et al*. in preparation). Briefly, barcoded DNA libraries for long-read sequencing were prepared using the ligation sequencing kit [SQK-LSK109, Oxford Nanopore Technologies (ONT)] and the Native Barcoding Expansion 1–12 (EXP-NBD104) kit. Libraries were sequenced for 24 h with R9.4.1 flow cells and an ONT GridION. Trace files were demultiplexed and basecalled using ONT Dorado basecaller (version 0.4.1) with high-accuracy basecalling. Raw ONT and Illumina read data were combined and assembled using Unicycler version 0.5.1 [20]. Long-read coverage of the genomes ranged from 43 to 360× coverage. Raw reads were submitted to ENA and complete hybrid genome sequences were deposited on *Neisseria* PubMLST (Data File S1). Whole-genome assemblies were downloaded from the PubMLST database in fasta format along with 11 reference genomes in GenBank format (Data File S1) [21]. A custom *Neisseria* genus database was created from the 11 reference genomes by applying the ‘prokka-genbank_to_fasta_db’ function in Prokka. Prokka (version 1.14.6) was then invoked to annotate the whole-genome assemblies with the custom *Neisseria* database as reference (prokka --genus Neisseria; --usegenus; --proteins; --addgenes; --centre) [22].

### Whole-genome analyses

Whole-genome alignment of the pan- and core-genomes was constructed from the prokka-outputted GFF3 files with PIRATE version 1.0.5 using default settings and specifying --align [23]. The core genome was defined as genes present in at least 95% of isolates. Individual alignments for *csw* were also produced in Roary using the query_pan_genome command with the -a gene_multifasta and -n arguments set. Maximum-likelihood trees of the core- and pan-genome alignments were generated using the GTRGAMMA model in RaxML-NG version 1.2.2 [24]. The ‘-f a’ and ‘-# 100’ arguments were specified to perform rapid bootstrap analysis, followed by a maximum-likelihood search, on the original alignment using 100 replicates. A core genome tree of 1,466 international MenW:cc11 isolates was produced using the MicroReact plugin on PubMLST with the cgMLST v3.0 scheme selected. The number of international isolates was reduced to fit within the limits of the MicroReact plugin by including only those isolated from 2017 onwards. The 55 pandemic/post-pandemic UK MenW:cc11 isolates that were contained within one of the SCs were also included in the tree. Trees were visualized using MicroReact [25]. Metadata plotted on the trees was collected from PubMLST (Data File S1). Groups were created from year of isolation data with these groups generally corresponding to significant events in the historical epidemiology of cc23 (Table 1). Allele numbers for specific genes were obtained from PubMLST.

**Table 1.** Rationale and isolate numbers for MenW:cc11 year groups

Year group	Event	No. of isolates^*^
1970–1979	Arbitrary 10-year time span	17
1980–1989	Arbitrary 10-year time span	3
1990–1999	Arbitrary 10-year time span	14
2000–2008	2000 Hajj outbreak up to 2009 outbreak in the UK	126
2009–2013	2009 outbreak in the UK up to clonal expansion in late 2013	186
2014–2019	Late 2013 clonal expansion until COVID-19 outbreak	1,101
2020–2022	COVID-19 pandemic	39
2023–2024	Post-pandemic	34

*Only 1,520 isolates are represented in the table as year of isolation data was lacking for one isolate.

### Bioinformatic analysis of *fetA* and *csw* gene sequences

The *fetA* and *csw* gene sequences for the above set of selected isolates were downloaded from the *Neisseria* PubMLST database. These gene sequences encompass the ORF from the ATG to the stop codon for a defined sequence (allele 1), which usually encodes the full-length protein. These gene sequences were aligned using mafft version 7.525 with the ‘--auto’ option [26]. A phylogenetic tree was inferred for each alignment using maximum likelihood (GTRGAMMA model) with Felsenstein bootstrapping in RaxML-NG [24]. For the csw phylogeny, a five-star GTR model was specified with the arguement - model MULTI5_GTR+Mi{ACGT-}{N} - to allow for indels to be factored into phylogeny construction. The *csw* tree was visualized in the same manner as the pan-genome tree. The *fetA* tree was visualized and managed using the Interactive Tree Of Life (iTOL) online tool [27]. As *fetA* can be subject to PV due to an SSR within the promoter region, flanking sequences for this gene were downloaded from the PubMLST database. Incomplete or missing sequences for the *fetA* intergenic region (IGR) and repeat tract were reassembled from raw reads by selecting an adjacent conserved 1 kb trap sequence from the N222.2 reference genome and mapping the pair-end reads to this sequence followed by reassembly with SPAdes [28,29]. The sequences were then aligned using the mafft online tool and analysed using BioEdit version 7.2.6 [26,30]. Evolution of the repeat tract over time was visualized using ggplot2 in R software version 4.4.0 [31,32].

## Results

### Post-pandemic expansion of SCs of MenW:cc11 lineages

**Fig. 1.**
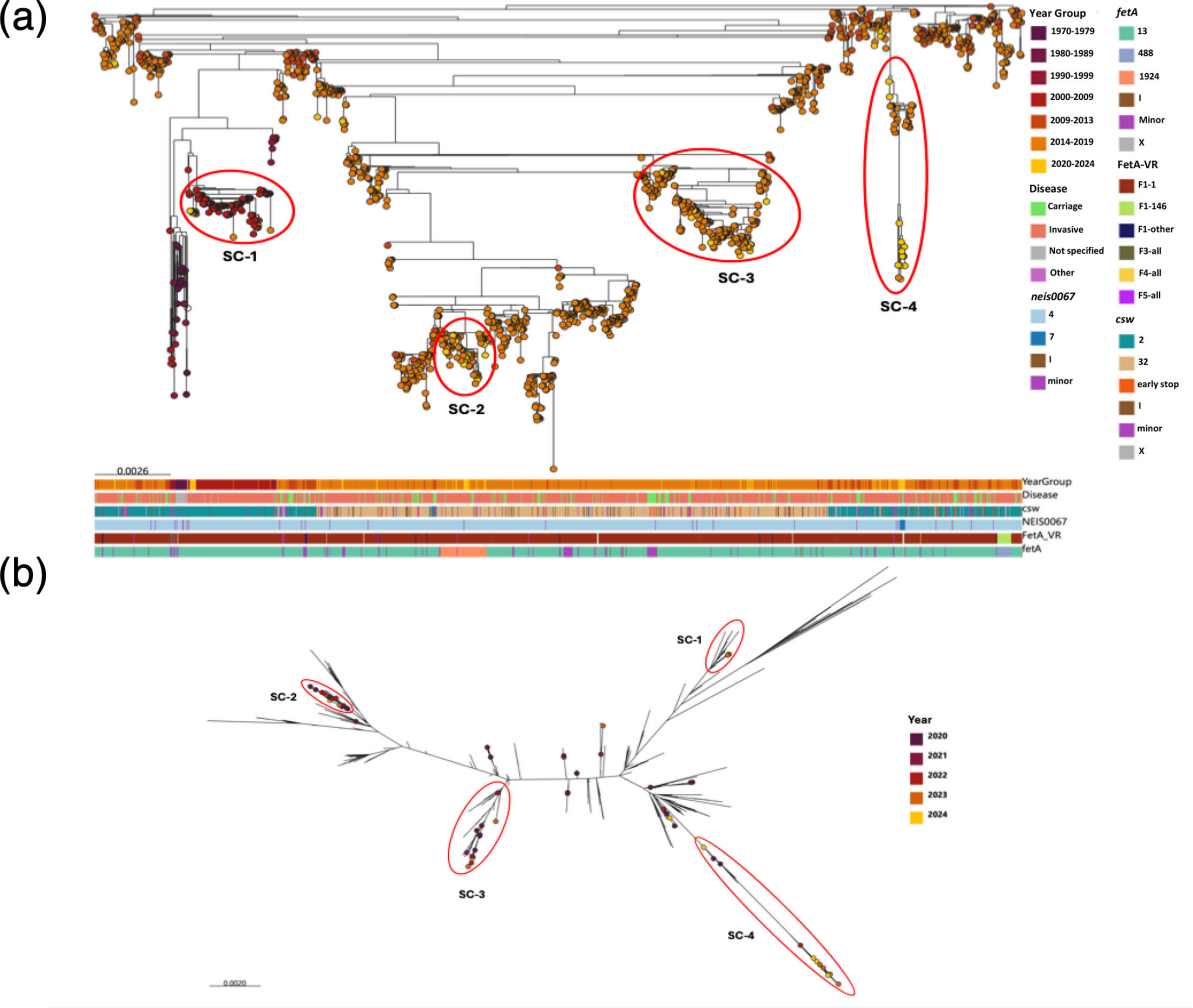
Maximum likelihood pangenome tree of 1521 UK MenW:cc11 isolates. This phylogenetic tree was constructed in RAxML-NG using a pangenome alignment. (**a**) Nodes are coloured based on groupings of the year of isolation as described in Methods and shown as year group in the legend. Horizontal bars are coloured as per the legend to display metadata for each isolate on the tree with the following classifiers: year group, disease state and major alleles for *csw* (capsule synthetase), NEIS0067 (capsule translocase), *fetA* VR (*fetA*_VR) and *fetA*. See main text. Minor alleles have been grouped (‘minor’), and alleles producing an early-terminated capsule synthetase form an additional group (‘early stop’). Labels X and I for genes indicate missing and incomplete sequences, respectively. The scale bar denotes the mean number of nucleotide substitutions per site. (**b**) Radial formatted tree highlighting only 2020–2024 isolates coloured by year of isolation, with SCs highlighted.

To explore how the epidemiology of UK MenW:cc11 isolates has changed over time and as a result of the COVID-19 pandemic, we examined the phylogenetic relationships in relation to arbitrary 10-year groupings or major changes in the spread of this clonal complex (Table 1). To assess the effects of the pandemic, we split isolates into two groups: pre-pandemic group, years 1974–2019, 1447 isolates; and a combined pandemic and post-pandemic group, years 2020 to 2024, 73 isolates. A pangenome alignment was created in PIRATE, which identified 6,190 gene families, of which 1,798 were present in >95% of isolates. This pangenome alignment was inputted into RAxML to produce a pangenome tree from 1,521 isolates. Isolates clustered strongly by year of isolation with pre-2000 isolates being mainly located in one part of the tree (see 1970–1979, 1980–1989 and 1990–1999 groups; Fig. 1a). The central part of the tree contained the 2013 sublineage (as characterized by having *csw* allele 32) from the periods immediately pre- and pandemic/post-pandemic (i.e. 2014–2019 and 2020–2024, respectively). A key feature of this UK pangenome tree is the presence of multiple, divergent phylogenetic branches (Fig. 1b). We have defined four SCs based on recognizably distinct branches within this tree that contain 75%(55/73) of the pandemic/post-pandemic isolates with SC1, SC2, SC3 and SC4 having 14, 25, 18 and 19% of these isolates, respectively. These SCs contain 458 isolates in total and have varying degrees of internal genetic diversity and isolate numbers. A significantly disproportionate number of these isolates were post-pandemic (55/458 within SC1-4; 18/1,063 outside of these SCs; chi-squared (1, *N*=1,521) 75, *P*<0.00001; https://www.socscistatistics.com/). SC-1 and -4 are from the original UK sublineage of MenW:cc11 (*csw*, allele 2) with the former containing some of the oldest isolates in the database (isolation years, 1980–1989). Notably, SC-4 (*n*=40) includes a large cluster (*n*=14) of pandemic/post-pandemic isolates that are near-identical on this pangenome tree (Fig. 1a) and are identical on a core genome tree (data not shown). These isolates co-cluster with 26 highly genetically similar isolates in SC-4 that were circulating in the 3 years immediately prior to the pandemic. The central portion of the tree contains 2013 sublineage isolates and the highest number of pre- and post-pandemic isolates but has some evidence of two divergent groupings (SC-2 and SC-3). Carriage isolates were limited in number and generally spread throughout the tree, indicative of random sampling of all major disease-associated groupings and of the close linkage of disease and asymptomatic carriage.

**Fig. 2.**
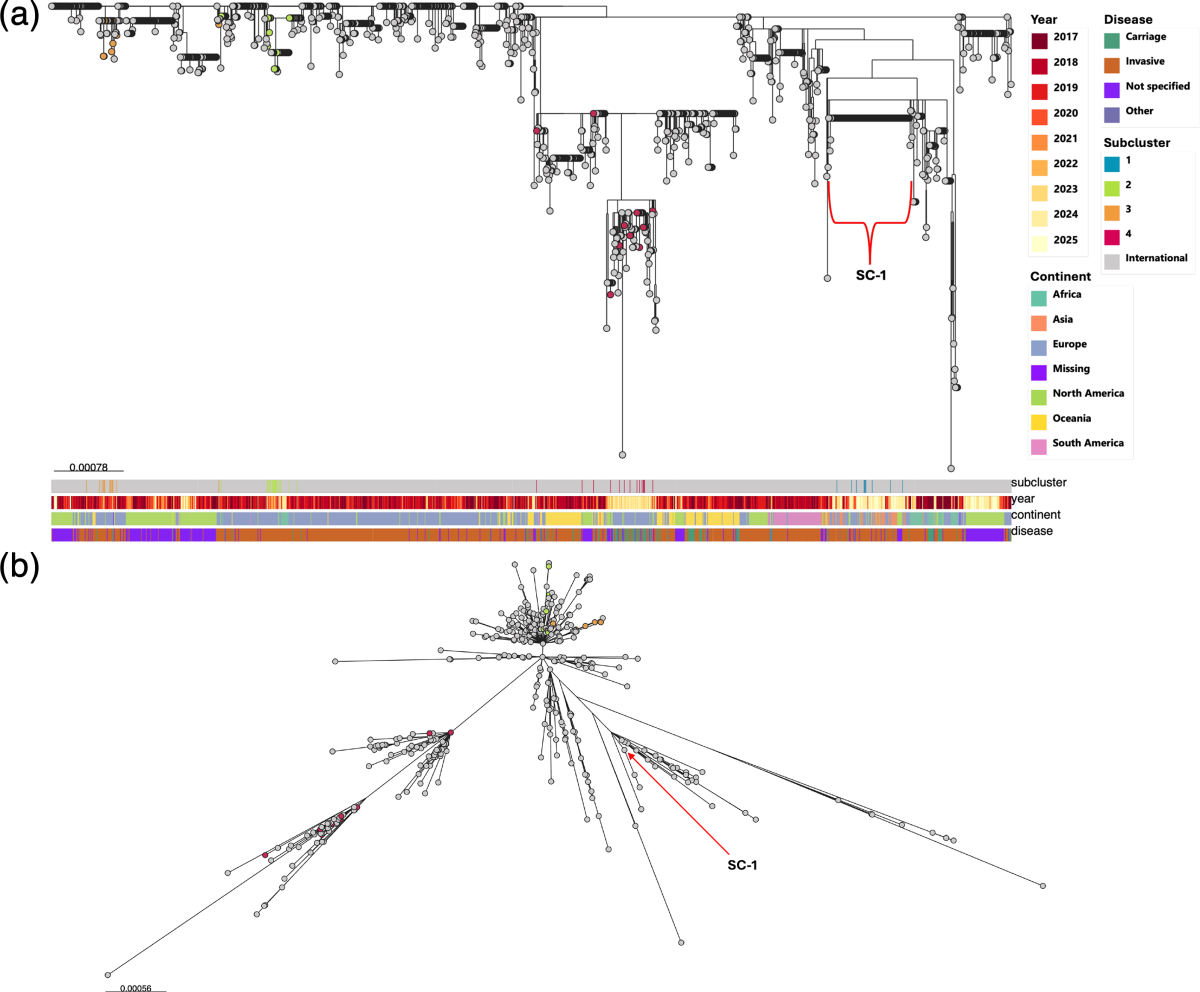
Neighbour-joining core genome tree of 1,466 international MenW:cc11 isolates and 55 pandemic/post-pandemic SC UK isolates. The phylogenetic tree was created on *Neisseria* PubMLST using the MicroReact plugin and selecting the cgMLST version 3.0 scheme. UK SC isolate nodes are coloured by the SC each isolate belonged to (see Fig. 1), whilst the 1,466 international isolates are coloured grey. Horizontal bars are coloured as per the legend and display metadata for each isolate for SC, year of isolation, continent of isolation and disease state. The scale bar depicts the mean number of nucleotide substitutions per site. (a) Dendrogram-formatted tree. (b) Radial formatted tree. The red bracket and red line indicate the location on the tree of UK MenW:cc11 pandemic/post-pandemic isolates belonging to SC-1 which were highly closely related on these trees.

To assess the global context of the UK pandemic/post-pandemic isolates, we compared these isolates to a worldwide collection of 1466 MenW:cc11 isolates for the years 2017 to 2025 in a core genome multi-locus sequence type (cgMLST) phylogenetic tree (Fig. 2). The reduction in genetic diversity of this tree resulted in SC-2 and SC-3 being co-clustered, reflecting high similarity in the core genome. The UK pandemic/post-pandemic isolates are co-located in clusters with isolates from other geographic regions, indicating that expansions of these SCs were occurring in a wider geographic region. Notably, SC-1 and SC-4 were responsible for most post-pandemic IMD cases, with SC-1 occurring in Europe, Asia and North America. SC-4 was mainly restricted to Europe with a few cases in North America. The large post-pandemic expansion of a different SC of isolates in North America but not the UK or other regions is intriguing but unclear as the disease determinant data was missing from the database and hence may represent carriage not IMD.

### Limited evolution of capsular biosynthetic genes

Previous work on UK MenW:cc11 isolates had detected evidence of strong associations between genetic variants of *csw* and virulence-associated phenotypes. The pangenome phylogenetic tree was, therefore, examined for evidence of allelic variants of this and other capsular biosynthetic genes (Fig. 1). As expected, the tree splits very strictly based on the two major alleles of *csw*, *csw-2* and *csw-32*, which delineate the previously observed original and 2013 MenW:cc11 sublineages with, as described above, the post-pandemic SCs being correlated with specific alleles. DNA sequence alignment showed that the two major alleles, *csw-2* and *csw-32*, differ by only three adjacent nucleotides spanning two codons but resulting in a single amino acid change from a negatively charged glutamine to a positively charged arginine (Fig. S1). Inspection of an AlphaFold structure, available on UniProt, for *csw-2* showed that the affected residue, E202, is proximal to the active site of the N-terminal domain, which is marked by the presence of an EX_7_E motif (Fig. S1).

**Fig. 3.**
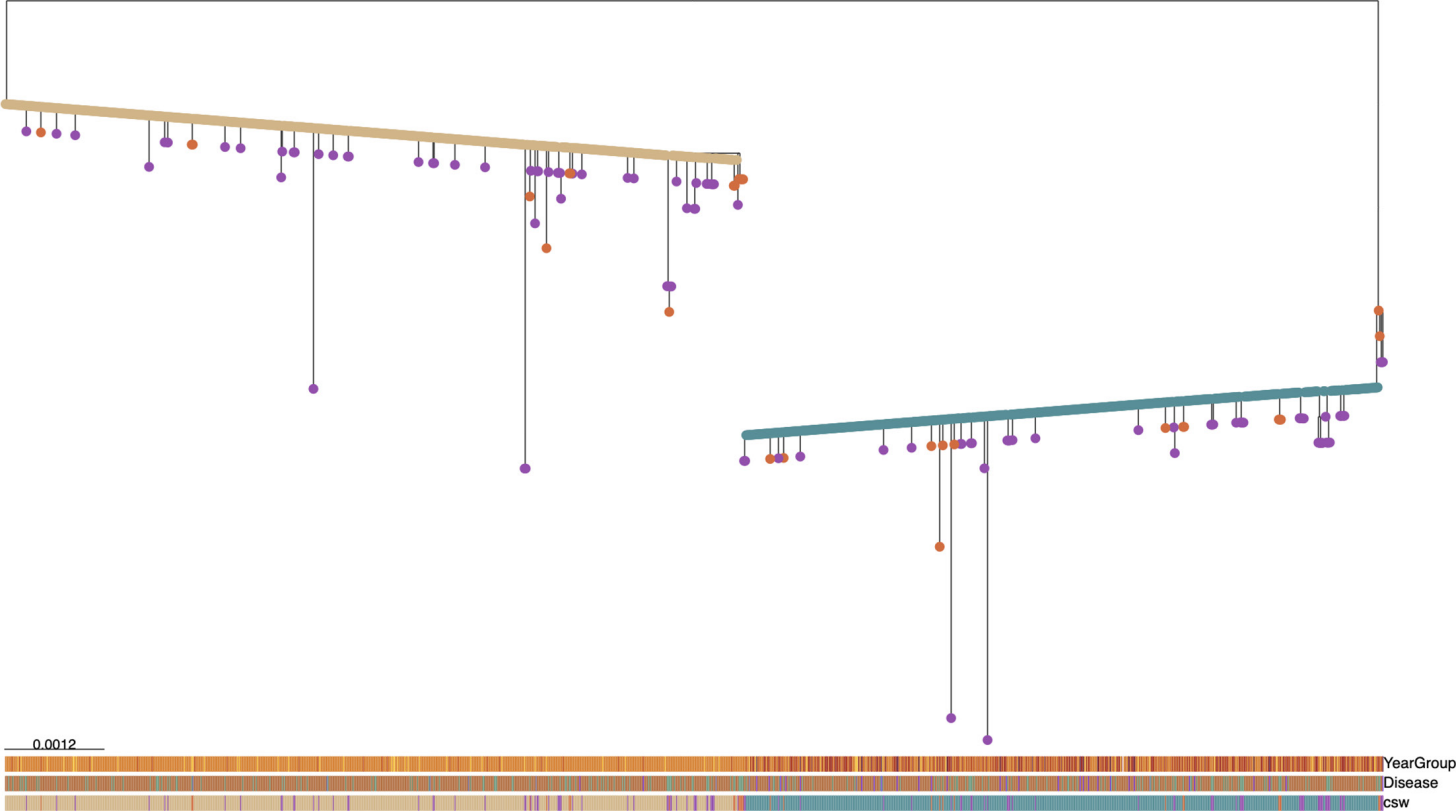
Maximum-likelihood tree of 1,325 sequences of the *csw* gene of UK MenW:cc11 isolates. This tree was generated from an alignment of intact *csw* gene sequences using RAxML-NG and modified to allow for indels to be factored into the phylogeny. A set of 196 isolates was excluded from this analysis due to either missing or incomplete *csw* gene sequences. Gene sequence data was obtained from PubMLST (see Methods). Nodes are coloured according to the allele of *csw* possessed by each isolate (major alleles 2 and 32, green and beige circles, respectively; early stop, orange circles, 19 alleles with an indel that results in an early STOP codon within the sequence; minor, 68 minor alleles, purple circles, that produced a complete protein). Horizontal bars are coloured as per the legend for Fig. 1 and show metadata for each isolate in the tree as follows: disease state, seven groups for year of isolation (year group) and *csw* allele. Scale bar denotes the mean number of nucleotide substitutions per site.

To further investigate minor *csw* alleles, a phylogenetic tree of 1521 *csw* gene sequences was constructed (Fig. 3). A total of 87 minor *csw* alleles were detected across 133 isolates, with most of these alleles differing from major alleles by one/two substitutions. The two major alleles, *csw-2* and *csw-32*, were present in 36.2% (*n*=550) and 42.2% (*n*=642) of these UK MenW:cc11 isolates, respectively (Table S1, Data File S1). Sequence data for *csw* was incomplete for 7.4% (*n*=113) and absent for 5.5% (*n*=83) of isolates. A set of 19 minor alleles had indels and substitutions that resulted in early stop codons and translational inactivation. These ‘early stop’ alleles were present in 29 isolates and were distributed throughout the population (Fig. 3). There was no obvious association between *csw* sequence and disease state apart from early stop alleles occurring almost exclusively (*n*=23/29, 79%) in carriage isolates, which themselves represented only a small proportion of the isolates (*n*=215/1,521, 14%). Of the remaining six isolates with indels within *csw*, four were invasive isolates and two lacked disease data. Interestingly, two of the invasive isolates were serogroupable, indicating that they were able to produce a capsule. Minor *csw* variants consisted of various substitution mutations and were almost completely absent among post-pandemic isolates. The minor alleles occurred more frequently in carriage isolates than invasive isolates, accounting for 14 and 6% of isolates in each group, respectively, suggesting a lower tolerance for *csw* variation among invasive isolates for which capsular expression is a critical virulence factor. Capsule expression metadata was lacking for many isolates possessing minor csw variants, with no recorded information for 22/104 isolates. Of the remaining isolates with minor *csw* variants, 77 expressed capsule (9 carriage, 67 invasive and 1 unknown) and 5 did not (4 carriage and 1 invasive).

Variation in other genes within the MenW-cc11 capsule locus was infrequent and randomly distributed throughout the phylogeny. One exception was *neis0067*, involved in capsule translocation, which had a divergent sequence in SC-3 isolates (Fig. 1; Data file S1; 15/1,260 and 4/419 nucleotide and amino acid substitutions, respectively).

### Evolution and spread of phase-variable *fetA* alleles

**Fig. 4.**
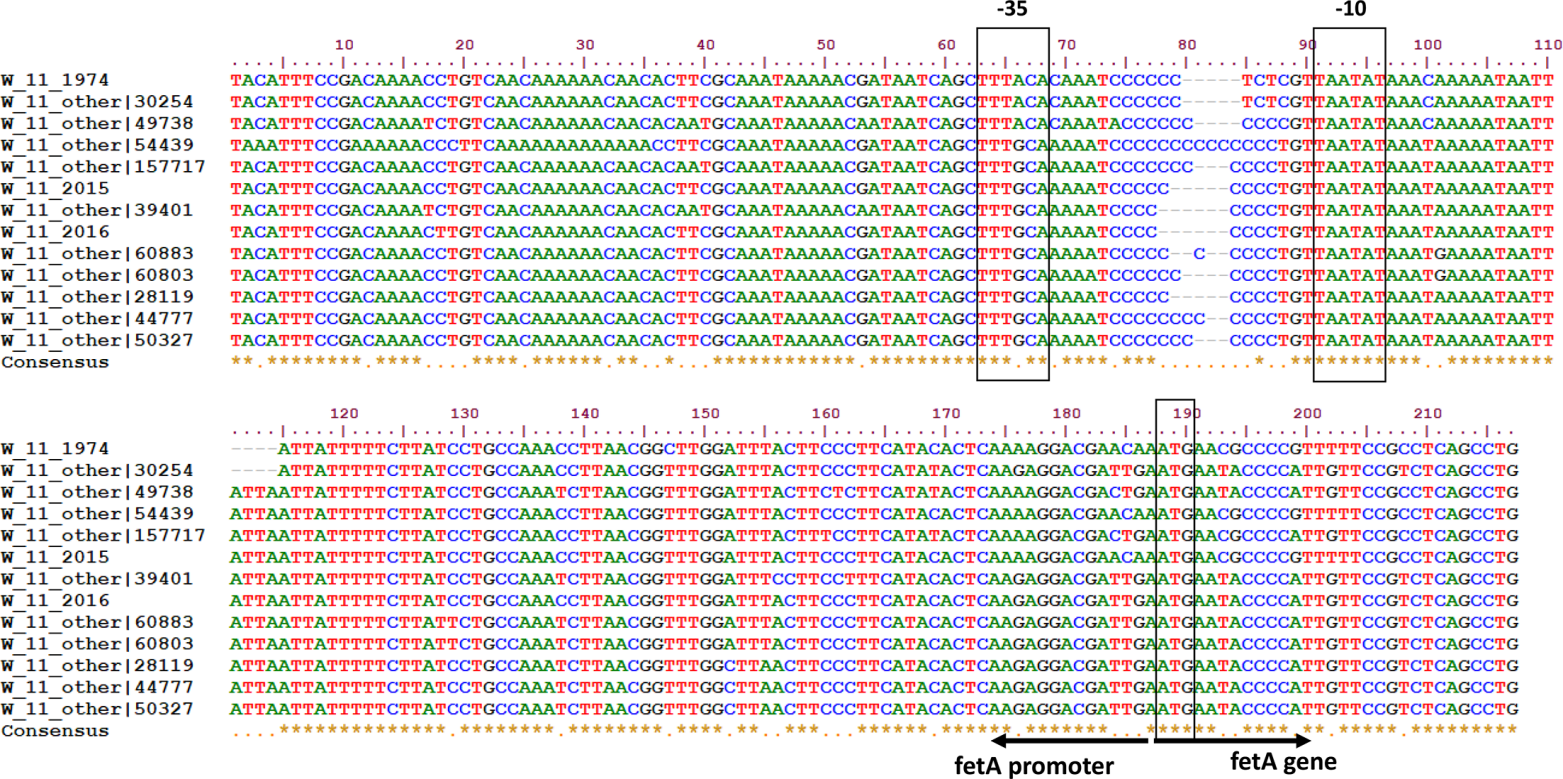
Variation in the polyC tract and core promoter of MenW:cc11 *fetA* promoter sequences. This figure shows the 13 different sequences obtained for 188 nucleotides of the *fetA* upstream IGR of 1,510 MenW:cc11 UK isolates. The three most common sequence types are W_11_1974, W_11_2015 and W_11_2016. Note that the W_11_1974 sequence has a 6C non-phase-variable tract. The other sequences were mainly found in single isolates and are minor variants of the major sequences. Boxed regions represent the presumptive -10 and -35 regions of the core promoter and the FetA initiation codon.

A previous study had shown that *fetA* was subject to PV in carriage, but not disease, isolates for a subset of MenW:cc11 isolates, indicating that *fetA* genetic variants might be a marker of carriage versus disease status. The allelic diversity of the complete gene, IGR and the hypervariable region (*fetA-VR*) was investigated. The *fetA* IGR was defined as the 188 nucleotides upstream of the ATG (see the W_11_1974 sequence in Fig. 4) as the sequences beyond this region contain a high prevalence of repetitive DNA. Rebuilding of the promoter region failed for 11 genome datasets, and hence, all data for these isolates were removed such that only 1,510 isolates were utilized in the *fetA* analyses. In contrast to *csw*, the vast majority of isolates contained the same VR (i.e. F1-1; 96%, 1,457/1,510) and gene allele (i.e. allele 13; 86%, 1,300/1,510). However, out of the 210 isolates that possessed unique *fetA* alleles, the majority were from the 2013 sublineage (59%, 123/210), with many of these isolates possessing either the 1924 (*n*=65) or 1254 (*n*=17) allele. Similarly, many of the original sublineage isolates possessed *fetA* allele 488 (*n*=17) and a novel VR (F1-146; *n*=17). Relative to allele 13, alleles 488, 1254 and 1924 had 1, 7 and 17 non-synonymous mutations, respectively. The tandem occurrence of both genic and VR variation was primarily in isolates obtained in the 2000–2008 period.

**Fig. 5.**
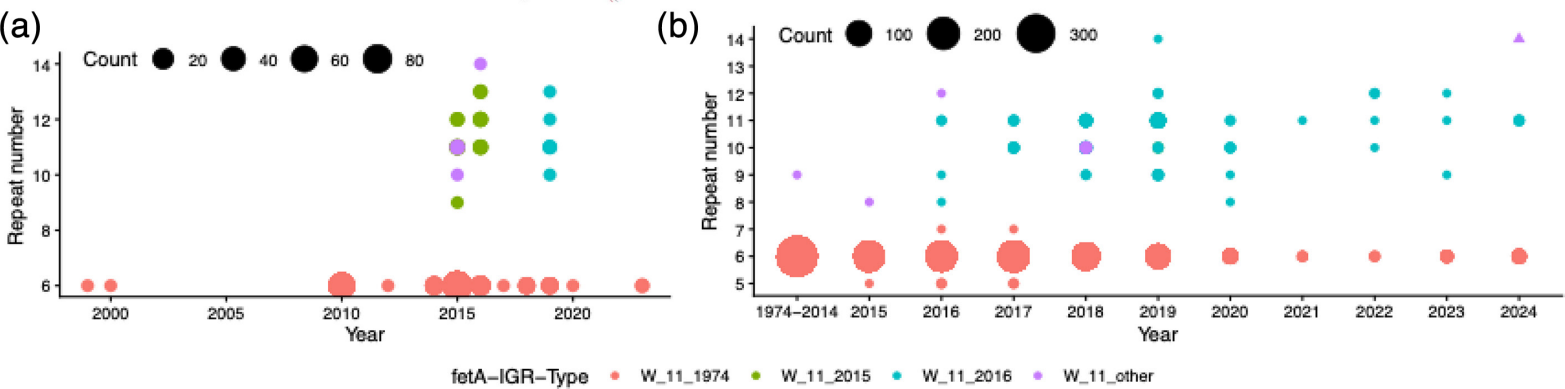
Evolution of the *fetA* polyC repeat tract between 1974 and 2024. This plot presents the evolution of the polyC tract of the *fetA* gene promoter for the UK MenW:cc11 isolates. (a) Carriage isolates (*n*=212). (b) Invasive isolates (invasive, *n*=1256, circles; other *n*=10, triangles). Note that the 32 isolates where strain source was ‘not specified’ were excluded as their disease phenotype was unknown. All 32 ‘not specified’ isolates had 6C tracts*.*

PV of *fetA* is due to an SSR located in the core promoter. Alignment of the *fetA* IGR sequences exhibited evidence of evolution of both core promoter elements and the polyC repeat tract (Fig. 4). To examine *fetA* IGR variation independently of PV, differences in the polyC tract were excluded, and then, each unique sequence was defined as a *fetA* IGR type. Most isolates contained one of three major sequence types: W_11_1974, W_11_2015 and W_11_2016. A small number of isolates contained unique sequences (labelled as W_11_other plus the identification number of the first isolate found to contain this sequence) with these sequences being minor variants of the major sequences. Previously, the MenW:cc11 *fetA* promoter was shown to have a 6C repeat tract (*fetA* IGR allele W_11_1974 in Fig. 4), which is not subject to PV. An increase to between 8 and 14Cs in this tract was detected for isolates obtained from 2015 onwards (Fig. 5). This increase in repeat number appears to have occurred due to a recombination event that stretches from nucleotides 65 to 115 in the shortest iteration (compare the W_11_1974 and W_11_2015 fetA IGR alleles) and replaced a 5′TCT sequence with 5′CCC thereby increasing the tract length from 6Cs to 9Cs (Fig. 4).

In order to examine the distribution of the different *fetA* IGR sequence types, isolates were classified as having one of the three major types (i.e. W_11_1974, W_11_2015 and W_11_2016 as shown in Fig. 4) or allocated to a minor allele group termed Other. These IGR sequence types excluded variation in the polyC tract, which was tracked relative to these groupings (Fig. 5). The W_11_1974 allele was present in all except one of the 463 isolates obtained between 1974 and 2014 inclusive. Of the 1,410 isolates containing the W_11_1974 allele, most (*n*=1403) had a 6C repeat tract, whilst seven exhibited one unit increases or decreases in repeat number exclusively among disease isolates (Fig. 5). The W_11_2015 sequence was almost exclusively (88%, 14/16) observed in carriage isolates obtained in 2015 and 2016 from a carriage study at the University of Nottingham (Fig. 5). This IGR sequence type was associated with variation in repeat numbers between 9C and 13C. The W_11_2016 sequence differs from the W_11_2015 sequence due mainly to alterations around the *fetA* initiation codon (Fig. 4). The W_11_2016 sequence increased in prevalence among invasive isolates from 2% (4/183) in 2016 to 21%(24/117) in 2019 and 29% (18/63) in the pandemic/post-pandemic period (Fig. 5). Other minor alleles were infrequent (*n*=9) and randomly distributed.

**Fig. 6.**
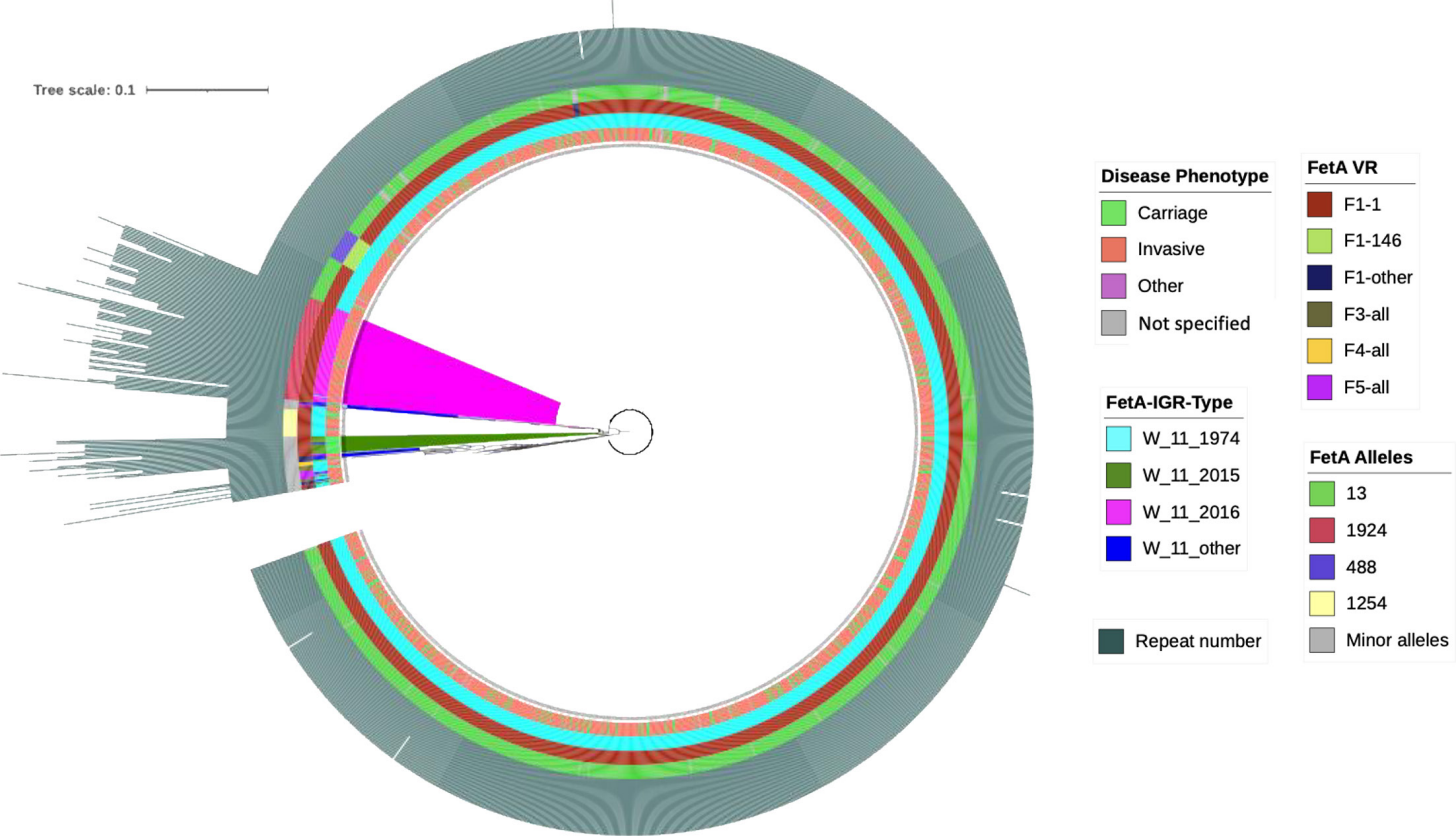
Coordinate variation in the *fetA* IGR sequence and repeat tracts but not VRs of UK MenW:cc11 isolates. This figure shows a maximum-likelihood tree derived from the intact *fetA* gene sequences for 1,510 MenW:cc11 isolates where allele number indicates a unique sequence as found in the *Neisseria* PubMLST database relative to the full-length ORF of a defined sequence (allele 1). The *fetA* IGR sequence types are as shown in Fig. 4. The FetA VR refers to a short amino acid sequence present on a surface-exposed loop of the FetA protein that is highly variable due to immune selective pressures [37]. This tree is annotated with four types of metadata: innermost ring, disease phenotype; second ring, *fetA* IGR type; third ring, *fetA* VR; fourth ring, FetA allele; and outermost ring, bar graph of the number of Cs in the *fetA* promoter repeat tract, range from 5 to 14. The highlighted clades show that the evolution of the repeat tract and IGR, but not VR, was linked.

A clear feature of the repeat analysis is that from 2016 to 2019, there was a significant increase among invasive isolates of *fetA* tracts of 8C or greater as compared to 6C tracts [3%–22%, respectively; 5/183 to 26/117; chi-squared (1, *N*=300) 29, *P*<0.00001; https://www.socscistatistics.com/]. The proportion of phase-variable repeat tracts was maintained and increased to 29% (18/63), among invasive pandemic/post-pandemic isolates, although this increase was not significant (chi-squared (1, *N*=181) 0.78, *P*=0.38) (Fig. 5). In order to determine whether the promoter variation was linked to *fetA* immunogenic variation, a maximum-likelihood phylogenetic tree was constructed from an alignment of 1510 intact *fetA* gene sequences of the MenW:CC11 UK isolates and annotated with *fetA* VR, IGR sequence type and repeat number (Fig. 6). The gene and IGR sequence types were stable in the majority of isolates, with *fetA* allele 13 being associated with VR F1-1 and the W_11_1974 IGR type (Data File S2; see also Fig. 1). The appearance of new *fetA* alleles with increases in repeat number was correlated with alterations in *fetA* IGR types but only to a limited extent with alterations in the *fetA* VR (i.e. most of these isolates retained FetA VR F1-1; Fig. 6).

## Discussion

The bottleneck imposed by the COVID-19 pandemic on many infectious diseases is likely to have affected genetic population structures. In this study, genetic diversity in UK isolates of the hypervirulent MenW:cc11 meningococcal lineage was examined for the pre- and post-pandemic periods with a focus on overall genome variability and variation in capsule, the major virulence determinant, and FetA, a highly immunogenic, phase-variable OMP. The post-pandemic period was dominated by four SCs of isolates, limited variation in *csw*, a capsular biosynthetic gene and high prevalence of a recently evolved phase-variable *fetA* gene alleles.

The COVID-19 pandemic and ensuing public health restrictions, most notably lockdowns, resulted in substantial decreases in IMD [33]. Removal of these measures presented opportunities for significant shifts in meningococcal epidemiology resulting from interruptions to transmission and reductions in natural and vaccine-acquired immunity. Post-pandemic IMD cases were characterized by a sharp rise in MenB disease but low rates for MenW:cc11 strains, a major cause of IMD prior to the pandemic [13]. Our data indicates that MenW cases are rising slowly with the emergence of four major MenW:cc11 IMD SCs in the pandemic (2020–2022) and post-pandemic (2023–2024) eras. Two of these clusters are closely related to pre-pandemic clones, but one SC (SC-4) has undergone a high degree of divergence. Another SC, SC-1, was closely related to some of the oldest MenW:cc11 isolates in this dataset, responsible for only a handful of IMD cases over the past 25 years in the UK. The association of post-pandemic disease with isolates from SC-1 suggests that the reservoir for the emergence of disease-causing strains, following periods of interrupted transmission, is broad and may be enhanced by waning immunity to rarely encountered isolates. We also observed broad overlap of UK post-pandemic isolates within SCs with isolates from around the world, suggesting that a more global shift in the MenW:cc11 population has occurred post-pandemic. However, European isolates dominated the available data, making up ~50% of all MenW:cc11 isolates deposited on PubMLST from 2017 onwards. Relatively few isolates originate from Asia or Africa, despite the prevalence of IMD in the meningitis belt in Sub-Saharan Africa.

The *csw* gene is the critical determinant for the synthesis of MenW capsules. Farzand *et al*. [5] had observed that indels in this gene were associated with loss of c.f.u. in heat-inactivated serum and also detected phenotypic differences between the original and 2013 sublineages potentially attributable to divergence in *csw* alleles between these lineages. Phylogenetic analysis of *csw* showed that indel alleles were distributed throughout the population, recapitulating findings from Farzand *et al*. [5], and indicative of the random occurrence of indel mutations during asymptomatic carriage of both sublineages (Fig. 3). Analysis of the *csw-2* and *csw-32* alleles identified a single amino acid substitution of opposite charge (E202R) that is outside of known active sites of the Csw protein. It is unclear whether this allelic variation alters functionality of the Csw N-terminal domain or explains functional differences between original and 2013 isolates [5]. The presence of similar proportions of both major *csw* alleles among post-pandemic isolates (*csw-2*, 37 %; *csw-32*, 47%) indicates that this allelic difference may not facilitate post-pandemic transmission of MenW:cc11 sublineages.

Analyses of the *fetA* promoter region detected proliferation of a phase-variable version of this sequence (the W_11_2016 *fetA* IGR sequence type), containing 8-14Cs, from 2016 onwards among invasive MenW:cc11 isolates. Other phase-variable *fetA* IGR sequences were detected prior to 2016 in two IMD isolates from 2012 and 2015. Intriguingly, the W_11_2015 *fetA* IGR allele was highly prevalent among carriers of a 2015–2016 carriage study but was not subsequently found among IMD isolates, which may indicate a chance occurrence among these carriers, a lack of fitness during disease or further recombination events (the W_11_2015 and W_11_2016 alleles differ in positions 183–208, on both sides of the initiation codon and by a point mutation at position 18 in the upstream region; Fig. 4). Whilst the pandemic lockdowns resulted in overall reductions of MenW:cc11 IMD, isolates carrying the W_11_2016 *fetA* IGR sequences were frequently observed among pandemic and post-pandemic MenW:cc11 IMD isolates.

The FetA protein can elicit type-specific bactericidal antibodies [34]. Whilst FetA is not a major component of the 4CMenB vaccine, evolution of *fetA* PV among MenW:cc11 isolates corresponds with the introduction of this vaccine into the UK routine infant immunization programme, suggesting that FetA PV may facilitate MenW:cc11 IMD in some 4CMenB-vaccinated individuals. Expression levels of FetA are likely to have been modified by alterations in the polyC repeat tract of this lineage with high expression correlating with 6C, 9C and 10C tracts, intermediate expression with 11 C and low expression with other lengths as detected for other lineages/serogroups [35]. Using these correlations as reference, FetA expression states for MenW:cc11 isolates with phase-variable genes are predicted to have the following distribution: high, 30 isolates (9C/10C); intermediate, 47 isolates (11C); and low, 20 isolates (8C/12–14C). High numbers of isolates with an intermediate expression state may suggest a balance between FetA function (siderophore-mediated iron acquisition) and immune evasion as observed for intermediate expression of the PorA protein in serum bactericidal assays [36].

In a broader view, the evolution of PV in the *fetA* gene mirrors observations of the evolution of PV in the *porA* gene and heightened PV of two Opa genes (due to increases in repeat number) in MenW:cc11 sublineage 2013 isolates from 2013 to 2017 [4]. Heightened PV of these three OMPs (FetA, PorA and Opa) may facilitate transmission of this lineage due to an enhanced propensity to escape adaptive immune responses during either longitudinal carriage or transmission to partially immune individuals.

## Supplementary material

10.1099/mgen.0.001655 Uncited Supplementary Material 1.

10.1099/mgen.0.001655 Uncited Supplementary Material 2.

10.1099/mgen.0.001655 Uncited Fig. S1.

## Abbreviations

cgMLST: core genome multi-locus sequence type
IGR: intergenic region
OMP: outer membrane protein
ONT: Oxford Nanopore Technologies
PV: phase variation
SSR: simple sequence repeat
VR: variable region
