# Supplementary material for: Rapid and recent evolution of fetA phase variability among hyperinvasive MenW:cc11 disease isolates
Source: Microb Genom. 2026 Feb 20;12(2):001655. doi: 10.1099/mgen.0.001655 (PMC13293289; doi:10.1099/mgen.0.001655)
Supplement: Uncited Supplementary Material 1. [file mgen-12-01655-s001.pdf]

Table S1. Frequencies and breakdown of *csw* alleles

Group	*n* isolates	List of alleles within group (*n* isolates)
2	550	2
32	642	32
minor	104	1 (1), 101 (1), 102 (2), 103 (1), 104 (1), 105 (2), 106 (1), 107 (1), 108 (4), 109 (1), 110 (4), 111 (1), 112 (1), 128 (1), 129 (1), 130 (1), 131 (1), 132 (1), 137 (3), 138 (1), 160 (1), 17 (2), 19 (2), 20 (5), 22 (4), 23 (2), 24 (1), 30 (2), 62 (3), 64 (1), 68 (2), 71 (1), 72 (1), 80 (1), 81 (2), 82 (1), 85 (2), 9 (1), 94 (1), 97 (1), 99 (1), new#1 (1), new#10 (1), new#11 (1), new#12 (1), new#13 (1), new#14 (2), new#16 (1), new#17 (1), new#18 (1), new#2 (1), new#20 (1), new#21 (1), new#22 (1), new#23 (2), new#24 (1), new#25 (1), new#26 (1), new#27 (1), new#28 (2), new#29 (1), new#30 (1), new#31 (2), new#36 (1), new#5 (4), new#6 (4), new#7 (1), new#8 (1)
early stop	29	118 (1), 134 (1), 25 (1), 26 (1), 27 (1), 28 (1), 29 (3), 70 (1), 78 (2), 79 (2), new#15 (1), new#19 (1), new#3 (2), new#32 (1), new#33 (2), new#34 (1), new#35 (1), new#4 (5), new#9 (1)
X[1]	83	-
I[1]	113	-

[1]X - missing sequence; I - incomplete sequence.

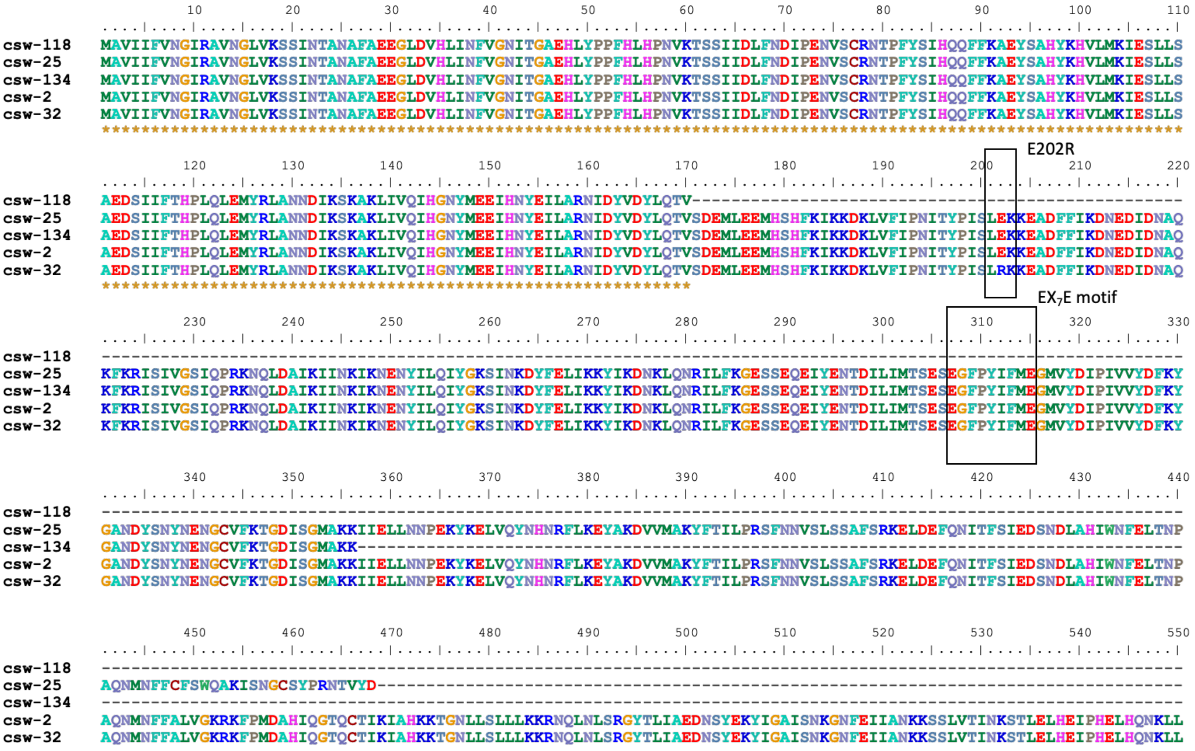

Figure S1. Alignment of the amino acid sequences of the major and truncated *csw* alleles of MenW:cc11 isolates. The csw-2 and csw-32 alleles are alleles of *csw* present in the majority of the 1521 UK MenW:cc11 isolates subject to genome sequencing between 1974 and 2024. The csw-118, csw-25 and csw-134 alleles contain insertions or deletions in their nucleotide sequences that result in truncation of the amino acid sequences as shown. Box 1 indicates the single amino acid difference between the two major arrows. Box 2 indicates the location of the EX_7E motif, the major active site of the Csw protein. Amino acid sequences are shown as single amino acid codes, dashes indicate missing amino acids due to truncations.
